# FAIR+E pathogen data for surveillance and research: lessons from COVID-19

**DOI:** 10.3389/fpubh.2023.1289945

**Published:** 2023-11-21

**Authors:** Aitana Neves, Isabel Cuesta, Erik Hjerde, Terje Klemetsen, David Salgado, Jacques van Helden, Nadim Rahman, Nazeefa Fatima, Nestoras Karathanasis, Pawel Zmora, Wolmar Nyberg Åkerström, Sushma Nagaraja Grellscheid, Zahra Waheed, Niklas Blomberg

**Affiliations:** ^1^SIB Swiss Institute of Bioinformatics, Clinical Bioinformatics, Geneva, Switzerland; ^2^Bioinformatics Unit, Institute of Health Carlos III, Madrid, Spain; ^3^Institute of Chemistry, The Arctic University of Norway, Tromsø, Norway; ^4^CNRS, Institut Français de Bioinformatique, IFB-core, UMS 3601, Evry, France; ^5^Aix-Marseille Univ, INSERM, Lab. Theory and Approaches of Genome Complexity (TAGC), Marseille, France; ^6^European Molecular Biology Laboratory, European Bioinformatics Institute, Wellcome Genome Campus, Hinxton, United Kingdom; ^7^ELIXIR Norway, Centre for Bioinformatics, University of Oslo, Oslo, Norway; ^8^Bioinformatics Department, The Cyprus Institute of Neurology and Genetics, Nicosia, Cyprus; ^9^Department of Molecular Virology, Institute of Bioorganic Chemistry Polish Academy of Sciences, Poznan, Poland; ^10^NBIS National Bioinformatics Infrastructure Sweden, SciLifeLab, Uppsala University, Uppsala, Sweden; ^11^ELIXIR Norway, Department of Informatics, University of Bergen, Bergen, Norway; ^12^ELIXIR Hub, Wellcome Genome Campus, Cambridge, United Kingdom

**Keywords:** data sharing and re-use, FAIR (Findable Accessible Interoperable and Reusable) principles, equity, COVID-19, surveillance

## Abstract

The COVID-19 pandemic has exemplified the importance of interoperable and equitable data sharing for global surveillance and to support research. While many challenges could be overcome, at least in some countries, many hurdles within the organizational, scientific, technical and cultural realms still remain to be tackled to be prepared for future threats. We propose to (i) continue supporting global efforts that have proven to be efficient and trustworthy toward addressing challenges in pathogen molecular data sharing; (ii) establish a distributed network of Pathogen Data Platforms to (a) ensure high quality data, metadata standardization and data analysis, (b) perform data brokering on behalf of data providers both for research and surveillance, (c) foster capacity building and continuous improvements, also for pandemic preparedness; (iii) establish an International One Health Pathogens Portal, connecting pathogen data isolated from various sources (human, animal, food, environment), in a truly One Health approach and following FAIR principles. To address these challenging endeavors, we have started an ELIXIR Focus Group where we invite all interested experts to join in a concerted, expert-driven effort toward sustaining and ensuring high-quality data for global surveillance and research.

## Introduction

High-throughput Sequencing (HTS) has made a huge impact in medicine, and pushed us into the era of personalized and genomic medicine. Microbiology is one of the fields where an unprecedented revolution has taken place, as HTS allows genomic characterization of pathogens of interest at clinical and public health levels, which eases their surveillance and outbreak control, making the concept of One *Health* (1, 2) a reality. Whole Genome Sequencing (WGS) technique has proven to be more informative and allows for better typing of microorganisms than classical techniques. The European Center for Disease Prevention and Control (ECDC) and the World Health Organization (WHO) have made recommendations to incorporate WGS for typing in outbreak surveillance and investigation at the global level, publishing notably a roadmap listing priority pathogens and deadlines for this analysis implementation (3, 4). HTS has many advantages such as high performance, quality, flexibility and scalability. HTS is gradually being applied to multiple tests carried out in a microbiology laboratory, such as the identification of microorganisms, outbreak characterizations and antimicrobial resistance determination, all essential for both microbiological surveillance and research.

The experience acquired by using WGS for bacterial outbreaks investigation allowed research and clinical laboratories to respond efficiently to the crisis provoked by the COVID-19 pandemic, where the sequencing of SARS-CoV-2 has contributed to enhanced diagnosis, treatment, vaccine development and viral evolution surveillance. The importance of viral genomic sequencing in clinical and epidemiological research is exemplified by the observed differences in speed and scale of genomic surveillance between the first acute respiratory syndrome (SARS) epidemic and the SARS-CoV-2 pandemic. Only 3 viral genomes were published in the first month of the SARS epidemic, reaching 31 in the following 3 months, representing valuable information for the molecular diagnostic yet not enough to follow viral genomic epidemiology in real-time at a large scale (5). On the contrary, during the COVID-19 pandemic, metagenomic sequencing allowed the identification of a new pathogen causing an unknown respiratory infection in just a few weeks in December 2019 (6, 7). Therefore, at the beginning of the year 2020, there were already hundreds of viral genomes in databases, currently reaching millions of sequences, setting a great example of a global effort on sequencing and data sharing. The sequencing of SARS-CoV-2 genome has proven to be an essential tool for the design of diagnostic PCRs, the study of outbreaks, understanding viral evolution and monitoring the effect of viral variants on the available vaccine or antiviral treatments. The viral genomic information has helped in taking public health measures, in accordance with the current epidemiological situation.

In 2021, the ECDC proposed possible public health measures to contain community transmission of the variants of interest (8) based on early detection of circulating variants by WGS of specific cases such as vulnerable patients, severe infections or cases from areas with circulation of variants of interest. To implement such measures, genomic sequencing had to be integrated into epidemiological surveillance. In the same direction, the European Commission urged member states to increase sequencing rates, targeting at least 5% of positive COVID-19 test results to be sequenced, to minimize delays from isolation to results and to ensure data sharing across countries, as active measures for surveillance (9).

**Figure 1:**
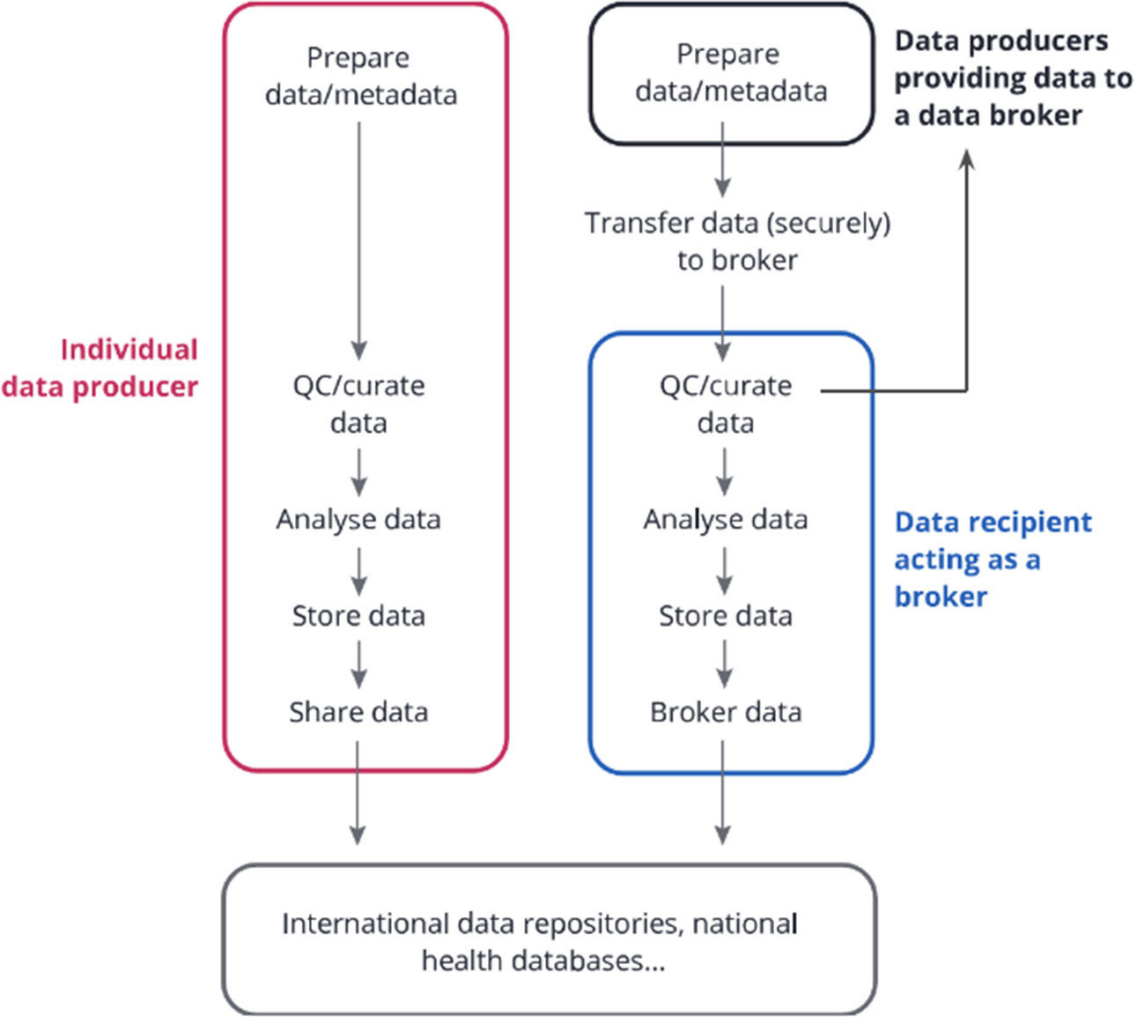
Data Brokering Workflow. Individual data producers can process the data, store it, and submit it directly to international repositories or public health databases. Alternatively, in the data brokering model, several data producers can submit their data to a common data recipient. This recipient might be responsible for curating the data, analyzing it with common pipelines, storing it, and re-sharing parts of the data to public health databases and international repositories (as agreed with the data providers), as well as ensuring data consistency and completion through close links and exchanges with the data producers. The latter service is often referred to as “data brokering” i.e. sharing data on behalf of others within a well-defined ethical and legal framework. Note that legal aspects should be considered along all the steps. Figure and legend modified with permission from (17) (CC BY 4.0).

The COVID-19 pandemic has exemplified the urge for international molecular data sharing together with minimal epidemiological metadata for interpretation. International data repositories have played a key role in enabling data access and reuse for research and surveillance through dashboards and epidemiological tools. Notable examples include the open EU Covid-19 Data Portal (10) and GISAID (11), as well as various data-enabled dashboards such as Nextstrain (12), CoVariants (13), CoVSpectrum (14), GalaxyProject SARS-CoV-2 analysis effort (15) and outbreak.info (16). For a successful data sharing process, the importance of data brokers has emerged in various regions and countries. This has proven to be an essential service to facilitate centralized data curation, standardized processing and re-sharing to various repositories with common anonymisation/pseudonymisation rules or to local public health authorities through tailored reports (Figure 1). In this model, individual laboratories perform pathogen characterization, then sequence or outsource sequencing to local/national sequencing platforms, and then submit their data and metadata within agreed standards to a local or national data hub responsible for data brokering to international repositories, thereby reducing duplication efforts across laboratories and fostering higher data quality, completeness and consistency. Such SARS-CoV-2 data brokering platforms have been successfully established in various regions and countries such as the UK (18), US, Germany, Denmark (19), Switzerland (20), Spain (21), Italy (22),^1^ France (23), Ireland, the Netherlands, Norway (24), Czech Republic (25), Poland (26, 27) and Austria (28), to support open data sharing.

The vast amount of data sharing (more than 10 million of consensus sequences deposited in databases such as GISAID or ENA), carried in part by these data platforms, demonstrate that many challenges on data quality and data sharing may have been successfully tackled, at least to some extent, by several countries. However, work remains to fully tackle challenges toward data sharing globally, in particular to ensure compliance with FAIR+E guidelines to make data truly Findable, Accessible, Interoperable and Reusable within an Equitable ecosystem. FAIR principles (29) have been very well-described. The addition of the Equitable concept (+E) to the FAIR principles was first introduced to our knowledge by WHO in their Guiding Principles for Pathogen Genome Data Sharing (30), where the authors acknowledge the need for further elaboration on how data sharing can support equity. From our perspective, a key element of equity relies on providing conditions that support the build-up of trust such as establishing data ownership where the data is generated, and through the implementation of an open community of practice of data sharing platforms, exchanging knowledge and expertise, by inclusively designing global data sharing architectures inviting diverse stakeholders to the table, and by embracing compromises in data sharing models (e.g. temporary access and re-use controlled data instead of immediate full open access data). The infrastructure, network and developed products now need to be maintained, anchored and further expanded to other data types such as wastewater and environmental datasets that have a great momentum to support global surveillance, bacterial datasets linked to antimicrobial resistance, and food-borne pathogens within a One Health context to enhance our understanding of infectious diseases and zoonotic infections, antimicrobial resistance, pathogen surveillance and outbreak response. It is absolutely necessary to share the genomic information at both national and international levels using well-aligned FAIR+E systems and governance. The data platforms also need to be more interfaced to avoid sensitive data silos and ensure that high-quality data is available for both research and surveillance. In the following section, we lay out the challenges that still remain and how the community could work toward addressing them all.

## Challenges

The WHO highlighted in 2018 the key challenges in establishing WGS for food-borne pathogens surveillance (30), divided into four categories: organizational, technical, scientific and cultural. The COVID-19 pandemic has represented a major proof-of-concept that WGS data can be used for global surveillance if timely data sharing challenges can be overcome. We discuss below the main challenges for using HTS SARS-CoV-2 data for surveillance and research, and present how some countries managed to address them, also in light of potential future threats.

### Organizational challenges

At the organizational level, coordinating national and global WGS-based SARS-CoV-2 surveillance required having access with the shortest time lag to the sequencing data generated within national surveillance and research programs. Durin g the COVID-19 pandemic, different countries started sequencing for surveillance at different times and with great differences in effort (31), resulting in spatial representativity biases that had to be accounted for when interpreting or downsampling the data for visualization purposes. In countries where the sequencing demand was high, data providers were often struggling to maintain routine diagnostics in parallel to running sequencing runs for surveillance. The data that was generated was then shared with international repositories for global surveillance and research [e.g. GISAID (11), EU COVID-19 Data Portal (10)] and with local public health authorities. In this context, many countries decided to establish national data hubs in order to avoid duplicating within each laboratory tasks related to data curation, data analysis and data entry/submission to multiple platforms, as well as to ensure the use of common standards and legal documents for data transfer, use and sharing for each envisioned application (e.g. Consortium Agreements and Data Transfer, Use and Processing Agreements). Such data hubs might also have a clear governance for data access for research purposes, facilitating data reuse within harmonized processes and accelerated ethical clearance. Through initiatives like ELIXIR CONVERGE (32), a pan-European network of SARS-CoV-2 data platforms has emerged where *ad hoc* practices in developing such data infrastructures and standardizing data and analysis workflows have been shared and discussed. Today, this community needs to be maintained and grow beyond COVID-19, and start implementing common good practices at the legal, ethical, organizational, scientific and technical levels.

Dedicated secured infrastructures such as Trusted Research Environments (TREs) were, however, often necessary to host such platforms, given the large amounts of data that were being produced and the fact that often sensitive metadata such as pseudonymised identifiers were also associated to enable linking to other datasets and hence avoid data silos. These sensitive data were generally not shared with the international community and remained within national silos, reflecting the need for a common trusted infrastructure enabling controlled data access and privacy-preserving queries such as the European Genomic Data Infrastructure (33).

While sequencing costs can be estimated quite accurately and were generally negotiated upfront, more rarely would (sufficient) budgets within national surveillance programs be allocated for data curation, analysis and sharing within a common data hub. While the funding for initially developing such platforms onto dedicated infrastructure was available in many countries, a survey performed within the ELIXIR network showed that in Autumn 2022, only 40% of the 11 surveyed platforms were fully funded for the year to come (unpublished), demonstrating the need to recognize better the costs incurred by digital platforms and for multiple organisms and funding bodies to engage at the national levels. As we enter more endemic times, the need for maintenance of these platforms should be recognized and we should seize the opportunity to expand the data types and features of such platforms to enable other applications such as surveillance of food-borne pathogens and antimicrobial resistance in a One Health context. Given the urgent context in which these data platforms had to be established, it is also key to take advantage of these more peaceful times to anchor efficient processes, good data management practices, automate as much as possible interfaces with data providers's laboratory information systems and refactor code where needed for increased robustness. Given the limited funding, consensus on prioritization should be made at the national and ideally supra-national levels.

### Scientific challenges

The COVID-19 pandemic has seen an unprecedented worldwide sequencing effort with more than 16 million consensus sequences submitted to GISAID (11) as of 17th October 2023. Sequencing volumes and time lag from collection to submission to international repositories varied greatly across countries and generally improved over the course of the pandemic (34). As an example, in August 2021, UK, at the time the shortest, had a median lag of 16 days (35) that went down to 10 days by the end of that year (36). Despite the global increase in sequencing capacity, a study from the CDC showed that disparities remained across economic lines for both these factors, advocating for increased geographic representation of virological surveillance and capacity building for increased timeliness of data submissions (36).

The large data volumes submitted to international repositories were key for global genomic surveillance that relied on high quality near-whole consensus sequences. Comparisons of genomic data quality using nextclade quality criteria suggest that Illumina-based sequences were of higher quality than nanopore-based sequences for the purpose of SARS-CoV-2 viral surveillance (37). It is interesting to note however that partial sequences can also be extremely useful to monitor predefined variants' prevalence from wastewater surveillance programs (38). The SARS-CoV-2 Data Hubs (39) are an example of bioinformatics tools for benchmarking, as the analysis produced a single large dataset of consensus sequences and variants from raw data via a pipeline developed by the Versatile emerging infectious disease observatory [VEO, (40)]. The Galaxy Project (15) and nf-core community (41) also proposed pipelines for SARS-CoV-2 analyses (15). The surveillance landscape would benefit from harmonized bioinformatics tools consisting of scripts, interfaces or application programming interfaces (APIs) readily available through open-source, documented and version-controlled repositories [e.g., GitHub (42), Gitlab (43)], and benchmarked against public datasets and through external quality assessment programs (44). Automation of routine analyses however does not mean doing without bioinformaticians and data managers, who keep playing a key role to ensure up-to-date analyses, scaling, and further investigation of more specific questions. In this context, the need to build capacity in bioinformatics and data management remains a challenge that ECDC is notably addressing by setting up dedicated training, also online (45). Software (source code, scripts, algorithms, computational workflows and executables) is essential to support scientific research and promote reproducibility. However, several challenges remain on the findability, accessibility, interoperability and reusability (FAIR) of software (46). Adaptation of these FAIR principles not only to data but also to research software is critical to enable harmonizing bioinformatics analysis and promote transparency and trust in scientific research.

In addition to high-quality data, genomic surveillance relies on associated high-quality metadata that adhere to common standards. Often disregarded, tremendous efforts in data curation have been deployed at local data hubs and international repositories to ensure metadata quality and integrity. Given the large sequencing volumes, this often required automating data validation processes with only minimal human intervention where required (47). The Public Health Alliance for Genomic Epidemiology (PHA4GE, https://pha4ge.org/) also developed a SARS-CoV-2 contextual data specification package (48) that notably supported data submitters and data brokers in mapping metadata to existing standards, and identifying minimal essential metadata and additional metadata that might be anonymized or access-controlled. The pathogen community however currently lacks a comprehensive ontology for pathogen genomics, as many relevant concepts are still missing from major ontologies [e.g. GenEpiO (49), SNOMED CT (50), LOINC (51)].

Data availability for research has a different meaning than for surveillance, yet it is key to support e.g., the development of new treatments, vaccines and a better understanding of viral biology and dynamics. In addition to consensus sequences, access to timely open raw data should be encouraged, avoiding embargos as much as possible. This will increase transparency, support reproducibility, and validation of results (52). Additional metadata can also be important for data re-use and reproducibility, describing the experimental setup with detailed protocols and including provenance reports on processed and analyzed data (using workflow management systems such as Snakemake (53) or Nextflow (54). Lastly, the pandemic has shown that sensitive metadata often remained siloed at data hubs. De-identified data might, however, preclude some studies to be conducted where e.g., datasets should be linked through a common, sensitive, sample identifier. The access to these data remains a great challenge even after the pandemic, mostly due to unclear legal frameworks, data governance and lack of international secure infrastructures to query and access these data.

### Technical challenges

The pandemic has highlighted the importance of generating, accessing and analyzing pathogen genomic data in near real-time for surveillance (variant tracking), diagnostic (PCR tests design), mitigation strategies (vaccine design, public health countermeasures) and research (vaccine discovery, antibody discovery, treatment development, viral biology etc.). Central to all this, national/regional data platforms were key to ensure that standardized and curated data of high quality were being shared to international repositories within the appropriate ethical and legal framework, reaching a wider audience such as public health experts and researchers, yet through a single point of entry for data providers. Some of the necessary technical components to build such infrastructures already existed and were expanded. To minimize the risk that countries and regions would operate as disconnected silos, an international effort was made in order to harmonize the work of establishing national and regional data hubs. The COMPARE data hubs (55) were notably expanded into SARS-CoV-2 data hubs, supported by several projects: https://www.covid19dataportal.org/partners?activeTab=Funding%20projects, as an essential component of the COVID-19 Data Portal (10). This enabled countries to organize, present and share their non-sensitive SARS-CoV-2 data with the international community, yet keeping sensitive data within separate national silos.

Some countries also expanded or developed their own platforms, in order to tackle specific tasks and activities for their users, such as variant reporting to public health authorities including sensitive data. In this context, the technical infrastructure was a key component, meant to be comprehensive and include data analysis, storage, sharing of sequence data and metadata, and analysis interpretations. In the SARS-CoV-2 genome sequencing effort, the IT challenges for a single country were typically related to human, compute and storage resources, as well as to sensitive data hosting and sharing within a highly secure IT infrastructure. HTS also posed technical challenges due to the growing diversity of sequencing platforms and the computational requirement involved, as well as the need for bioinformatics skills for downstream data analysis and its difficulties in standardization and harmonization. This required more work for assessing IT needs and for integrating dedicated analysis pipelines addressing diverse users' needs into regional/national platforms.

From a global perspective, each data platform was built differently and adapted to national and regional needs. Human resources and available technological solutions also added to the differentiation of the platforms. In general, only de-identified data was shared with international repositories, due to the lack of common agreement on how to find, access and share the sensitive part of the metadata. In order to make full use of sequence data, it would however be essential to be able to find data on any platform by e.g. setting up FAIR Data Points (29, 56, 57), a challenge addressed by only a few countries, and then have access to the contextual data, including patient clinical or epidemiological data that potentially can identify single individual persons. In this context, FAIR maturity indicators and automated solutions could be used to assess where improvements would be needed and support individual platforms in their FAIRification (58–60). Due to many ethical and legal constraints, the implementation of a sensitive data query system across various regional and national data platforms remains a challenge. The use of data and metadata standards would here be key to enable interoperability and quality standards for accurate comparison. Altogether, this would maximize the reuse of data and ensure that data follow FAIR principles. In human genomics, this challenge has been partly overcome through the Federated European Genome-phenome Archive [FEGA, (61)], where the data is archived nationally in Trusted Research Environments, and only the descriptions of datasets are available through federated searches using Beacons (62), with access to the data being granted by a Data Access Committee.

### Cultural challenges

Cultural differences result from different “standards” across countries and societies, as well as different national policies, e.g., on the SARS-CoV-2 surveillance. Regarding pathogen data, an important cultural challenge revolved around open science practices that differed greatly from one country to another. While everyone would agree on the necessity of timely data sharing, concerns about open data were rightfully invoked to ensure that data providers are properly cited and have time to perform their own research in a world where research benefits are not equitably distributed. This was particularly true for low to middle income countries (LMIC) and calls for flexible data sharing models including e.g. both timely data sharing to public health users under controlled access and reuse, and delayed (embargoed) access for researchers, making sure to also address Equity in the FAIR+E data sharing principles. More work is needed to address the operationalization of data sharing equity based on a global architecture and to define corresponding assessment metrics and benefit sharing mechanisms through global policy discussions such as those undertaken by the WHO on digital sequencing information and genetic sequence data (63).

The “publish or perish” aphorism also played a role in high-income countries (HIC) where researchers were not always keen to immediately release their data openly, even in contexts where research and data generation were funded by public money. In this regard, great differences across countries were observed and Ministries of Research and funding agencies have an important role to foster and raise awareness on open research data practices following FAIR principles. The COMPARE Data Hubs/SARS-CoV-2 Data Hubs (55) were developed to support open data sharing in these scenarios too—enabling for 'private, pre-publication' state for data. Yet overall, determining when data can be accessed and for what purpose remains a challenge for the international community, which needs to propose as well data sharing benefits for researchers such as citation or acknowledgment of credit for employment reviews and for promotions.

Differences in the amount of coordination and collaboration within a country also reflect cultural habits that can impact data generation and sharing within a pandemic context. Indeed, contexts where single-center studies are preferred over consortiums are highly prone to creating data silos. Discussing how scientists are rewarded within large consortiums remains a challenge to be clarified to ensure that key stakeholders are included in global efforts (and willing to do so).

Lastly, society's expectations of data availability, presentation and interpretation also differ across countries and have evolved during the pandemic. In the era of “fake news”, there is an urgent need to provide trusted sources of information and data, hosted or endorsed by trustworthy institutions.

## Solutions for addressing the remaining challenges

### Continue supporting global efforts toward addressing challenges in pathogen molecular data sharing

The Global Microbial Identifier Network—GMI (https://www.globalmicrobialidentifier.org/) consists of approximately 260 experts members from 50 countries, including clinical-, food-, and public health microbiologists and virologists, bioinformaticians, epidemiologists, representatives from funding agencies, data hosting systems, and policy makers from academia, public health, industry, governments, started in 2011, with the vision of developing a global system to aggregate, share, mine and use microbiological genomic data to address global public health and clinical challenges. GMI has been working on the challenges for global data sharing and emphasizing the need for quality through the establishment of several ring trials for quality assurance. In its next conference, GMI13 will focus on the critical importance of equity and interoperability (semantic, process, systems) in developing a global microbial genomics data sharing ecosystem (https://gmi13.org/).

As a result, the COMPARE Data Hubs have been developed (55). The Data Hubs system at the EMBL-EBI now continues to attempt to address and further support open data sharing and reduction in data silos. It does so by enabling groups to set up “COMPARE or SARS-CoV-2 Data Hubs” (55), supporting collaboration amongst users on data, data sharing, and potentially integrated analysis and visualizations, centring primarily around sequence data. Data can remain private until publication, or can be immediately public at the point of submission, offering a level of flexibility. This system aims to extend into general pathogens and preparedness, with greater automation and usability, and has linked with other biodata, including sensitive clinical-epidemiological data through cohort data sharing (64), a major benefit of sitting on top of EMBL-EBI infrastructure. The EMBL-EBI Pathogens Portal (65) enables finding and accessing data across the Data Hubs. Since all metadata associated to a sample are eventually openly published, only non-sensitive data can be collected at the pathogen data hub. This system also requires further development as mentioned as part of the package of extensions, and lacks the ability to pool mixed data together, e.g. via dedicated local/national TREs.

The US Food and Drug Administration has also established GenomeTrakr, a distributed network of laboratories using WGS for pathogen identification. All the collected data are stored in the publicly accessible GenomeTrack reference database, built initially for food-borne pathogens (66). Data curation and bioinformatics analyses are provided by the National Center for Biotechnology Information (NCBI) at the National Institutes of Health. Only non-sensitive data may be shared as all data immediately become publicly available.

The Public Health Alliance for Genomic Epidemiology (67) focuses on enabling FAIR public health bioinformatics including data standards, harmonization of tools and best practices documentation. It builds upon the work of five working groups on (i) Data Structures, (ii) Infrastructure, (iii) Bioinformatics Pipelines and Visualization, (iv) Training and Workforce development, and (v) Ethics and Data Sharing.

More recently, the WHO launched a call to host an International Pathogen Surveillance Network to accelerate pathogen genomics surveillance (68). An important aspect relevant here would be the creation of a Community of Practice on genomics data “to harmonize data standards and protocols, ensure genomics data tools are fit for purpose, and that data and benefits sharing are enhanced” to also embrace Equity. In this context, we anticipate that a global Community of Practice of local pathogen data platforms is a key element to enable an open forum, sharing of expertise and knowledge, and ultimately build trust and mutual understanding for setting up appropriate benefits sharing and data sharing models.

These concepts also exist in other contexts, such as the FEGA/CEGA for human data (69), the European Genomic Data Infrastructure (70) with also use cases in infectious diseases, the EJP-RD for rare diseases (71), or the PHIRI for population health (72).

Many scientists are involved in more than one of these initiatives (ELIXIR, GMI, PHA4GE, WHO IPSN), which will be essential to ensure that the challenges tackled by each initiative do not overlap or if so, benefit from complementary perspectives and mutual exchange of progress. Transversal working groups may be formed where relevant and representatives of each initiative should be invited for progress reports on topics of shared interest. Importantly, a global consortium bringing together members of all these initiatives and other important stakeholders should be established to implement Solution 3/below.

### Establish a global capacity building programme rooted in a distributed network of regional/national Pathogen Data Platforms

In order to address the remaining challenges while building upon all the valuable initiatives already in place, we propose to build capacity and extend a distributed network of regional/national Pathogen Data Platforms (PDP). Each PDP should be in close contact with local data providers, as these will be the main users and drivers of that PDP. The number of PDPs may vary from one country to another, with the aim to have as few as possible but as many as necessary given the local geopolitical health context of each country. The establishment of a regional/national PDP should aim to:

• **Ensure high-quality data, metadata standardization and data analysis**.

° Ensure timely collection of regional/national pathogen molecular data with internationally agreed quality metrics and minimal metadata.
° Structure data using controlled vocabularies (CV) and ontologies where they exist.
° Foster the establishment of data curation services within each PDP (set up common standards, share validation tools, etc.).
° Foster benchmarking of tools within reference datasets or through participation to External Quality Assessments.
° Implement common pathogen-specific bioinformatics pipelines across data providers and make the code publicly available.

• **Perform data brokering on behalf of data providers both for research and surveillance**.

**Figure 2:**
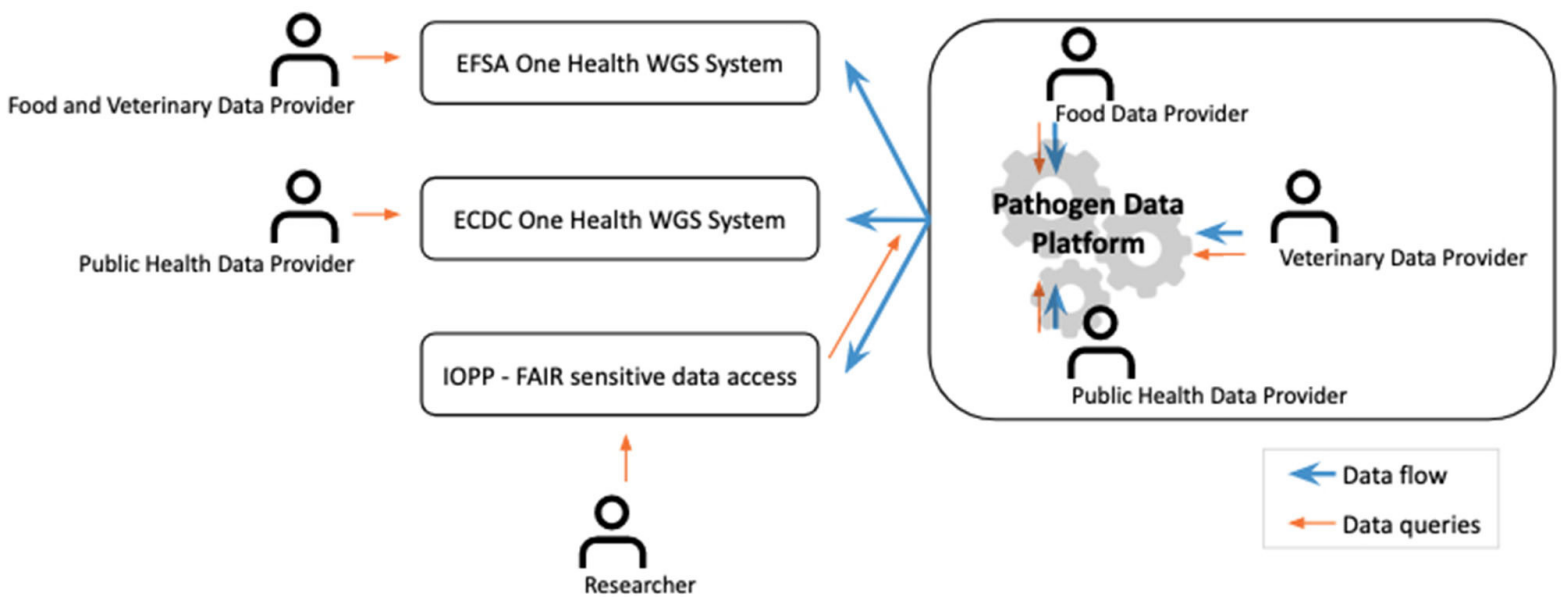
Integration of the PDP/IOPP infrastructures within the existing surveillance and research ecosystems. This cover has been designed using Gears icons created by Freepik from Flaticon.com.

° Reduce workload by being a single point of entry for data providers.
° Ensure that the collected data are shared within a well-defined ethical and legal framework common to all data providers.
° Promote FAIR sharing of data on domain-relevant international repositories.
° Promote open data sharing where possible, yet allowing each PDP to have its own policy or agree at the international level on embargo periods (e.g. LMIC vs. HIC).
° Become a trusted partner and data broker for global public health agencies such as the European Center for Disease Prevention and Control (ECDC) or the European Food Safety Agency (EFSA) WGS Systems, by preparing data complying with their requirements for the PDP's data providers (Figure 2).

• **Foster capacity building and continuous improvements, also for pandemic preparedness**.

° Build upon the ELIXIR Maturity Model (73) to support nascent and established PDPs in their development lifecycle. The Pathogen Data Platform Maturity Model consists of a set of 36 indicators to be evaluated when establishing or running a pathogen data platform. Develop open-source modular services to be integrated across PDPs.
° Develop, maintain and scale PDPs to support pandemic preparedness.
° Deliver trainings and documentation on essential aspects related to establishing and running PDPs such as data brokering (17, 74), data management, secure IT infrastructure, data sharing, ethical and legal aspects etc.
° Provide end-user support for all services provided by the PDP.
° Nurture trust between data providers and the PDP within well defined local governance and agreements.

### Establish an international one health pathogens portal

**Figure 3:**
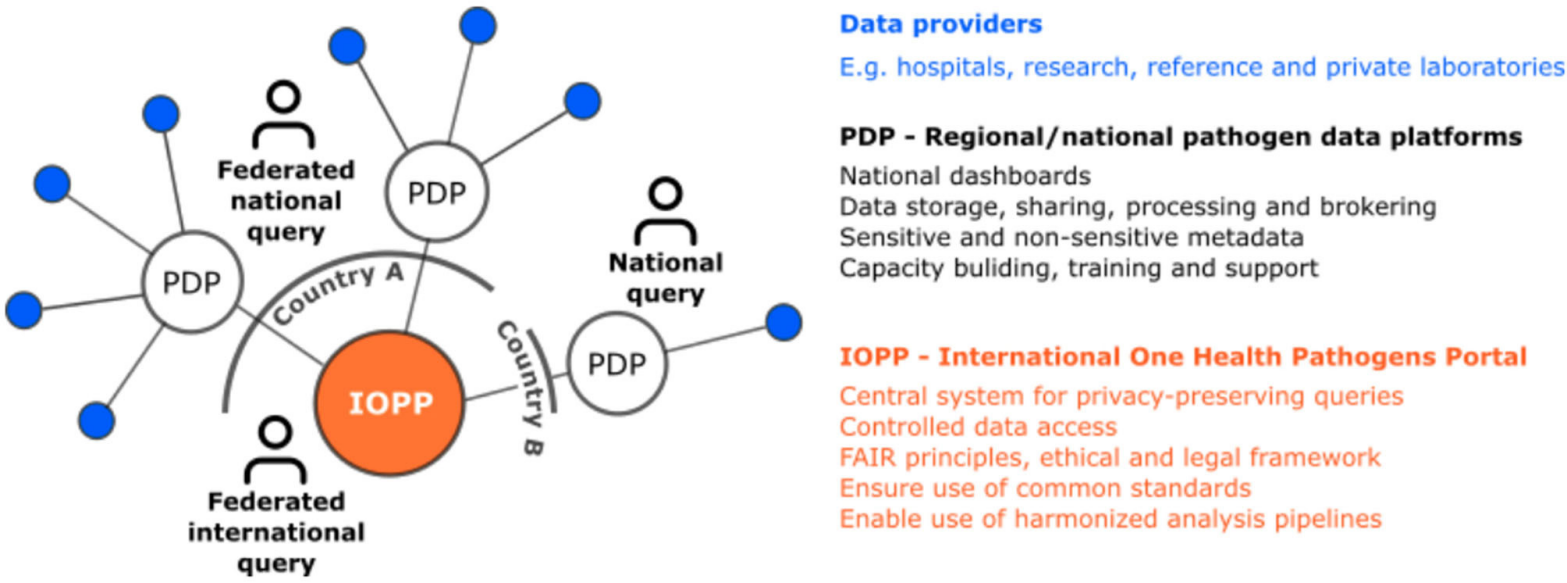
A distributed network of Pathogen Data Platforms for high quality research data and FAIR data access.

The distributed network of PDPs is also envisioned to enable FAIR+E data thanks to the transparent governance of an International One Health Pathogens Portal (IOPP) connected to each PDP and ensuring timely and equitable access to interoperable sensitive data. The IOPP would be established inclusively by putting together a global community representing all stakeholders and would follow FAIR+E principles within a well regulated ethical and legal framework (Figure 3), also ensuring interactions and mappings with other international repositories as recommended by others (75). It might be hosted by the European Bioinformatics Institute as an extension of its Pathogen Portal (65) to fulfill the requirements set out here and with joint governance by the PDPs. By connecting pathogen data isolated from various sources (human, animal, food, environment), the IOPP enables truly a One Health approach. It serves the following aims, through its coordination bodies:

- Enable privacy-preserving queries and support PDPs in establishing interfaces with the IOPP according to agreed standards.
- Control data access thanks to Data Access Committees, acting under a clearly regulated framework to also preserve data ownership. Support PDPs in labeling data with predefined access levels for semi-automated data access.
- Contribute to international standards definition, where needed. Support PDPs in adhering to common data standards.
- Foster open sharing of workflows and benchmarking with common open datasets.
- Organize pathogen/topic-specific workshops to harmonize analysis pipelines. Define quality labels for processed data generated within workflows successfully evaluated at External Quality Assessments programs. Harmonization of data production and metadata associated will contribute to useful data sharing.
- Define minimal standards for data, metadata description, including provenance reports for processed data.
- Encourage implementation of FAIR+E Data Points at each PDP.
- Establish differentiated data access and reuse rules for research and surveillance needs, taking into account different perspectives on open science and the need for benefit sharing.
- Promote equity, by ensuring that credit is given to data providers and processors through metadata requirements and appropriate citation procedures. Consider embargo periods or benefit-sharing conditions to be implemented.
- Support pandemic preparedness globally, by providing FAIR+E data to the international research community.

## Next steps for implementing the distributed network of PDPs and the IOPP

During the COVID-19 pandemic, several countries established regional/national SARS-CoV-2 data hubs based on the EMBL-EBI infrastructure or in-house developed, covering already part of the activities envisioned here for a PDP, to various levels of maturity. Existing infrastructures such as the EMBL-EBI Pathogens Portal also feature already many functionalities envisioned for the IOPP and might be extended to serve the other aims listed above.

Through the ELIXIR CONVERGE initiative (32), managers of nascent and established SARS-CoV-2 data hubs have been meeting on a regular basis to discuss and address common issues and needs, notably on data brokering to open data repositories. The concept of PDP/IOPP was born within this dynamic and collaborative working group who is now willing to expand and further collaborate to set the foundations of the IOPP and distributed network of PDPs.

**Figure 4:**
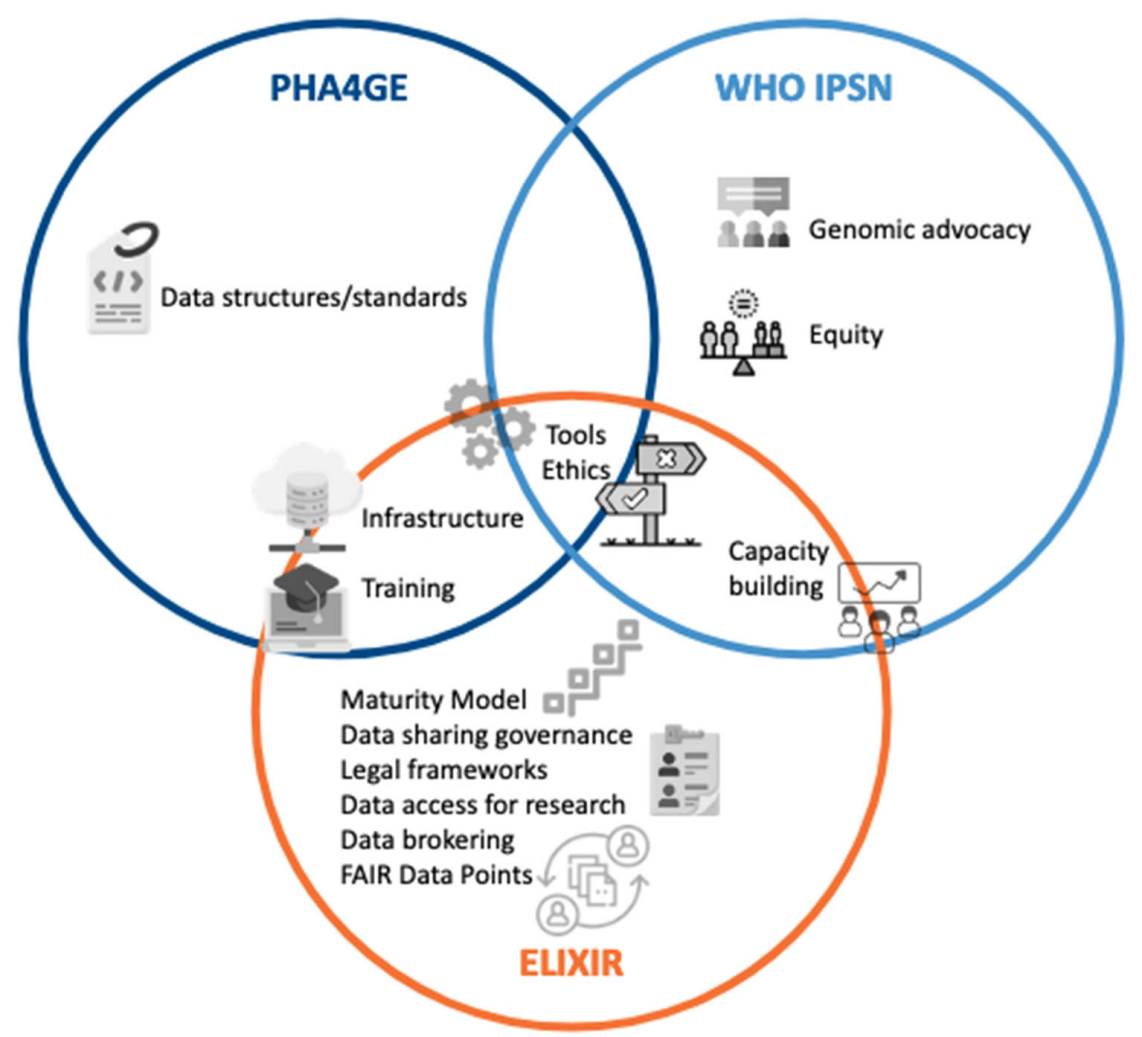
Complementarity of the various initiatives covering pathogen data sharing. This figure has been designed using images created from Flaticon.com (conversation by Freepik; justice by noomtah; hosting by Freepik, data-content-standard by Freepik; online-learning by Freepik; settings-gears by Freepik; choice by PopVectors; access-control-list by Freepik; levels by orvipiexel; collaboration by FreePik; efficiency by Mehwish).

To achieve this, an ELIXIR Focus Group on Pathogen Data will be established, with dedicated task forces to properly plan the PDP/IOPP roadmap and build/extend its infrastructure, governance, legal and ethical frameworks, Maturity models, data brokering, data access committees, interactions with surveillance authorities (EFSA, ECDC) and FAIR+E data in general (data standards, ontologies, CV, data brokering, open software, benefit-sharing). Worldwide experts are welcome to join this effort that will certainly occur in collaboration with other complementary initiatives (Figure 4). In view of pandemic preparedness and of the growing urgency in antimicrobial resistance in a One Health context, it is however essential that this network persists and grows into a stable infrastructure with a well-established ethical and legal framework as well as programming interfaces for efficient data searching and access across borders, with benefits-sharing ensured through the involvement and collaboration from key stakeholders from WHO (3). The aim of the Focus Group is to become an ELIXIR Community and global discussion space. This is an important step in order to gather experts, researchers and stakeholders to support this global work in establishing the foundations of the PDP/IOPP ecosystem with pilot implementations. Although our internal survey has revealed a great need for an international system to manage and share pathogen data for surveillance, the PDP/IOPP will only become successful if this is a collaborative effort. Hence, data providers, receivers and users need to have confidence in the system, and an ELIXIR Community can be an essential step to build this trust. As the WHO Director writes, “Three key principles repeatedly emerged during our discussions and should be seen as the basis of any future pandemic preparedness: trust, solidarity and equity, and sustainable development” (76). It is a unique opportunity to be seized now, to anchor and scale upon what has been built in the past years and use the lessons learnt for the future in a concerted, expert-driven global effort.

## Author contributions

AN: Conceptualization, Funding acquisition, Methodology, Project administration, Visualization, Writing – original draft, Writing – review & editing. IC: Conceptualization, Methodology, Project administration, Visualization, Writing – original draft, Writing – review & editing. EH: Conceptualization, Methodology, Project administration, Visualization, Writing – original draft, Writing – review & editing. TK: Conceptualization, Writing – review & editing. DS: Conceptualization, Writing – review & editing. JH: Conceptualization, Writing – review & editing. NR: Conceptualization, Writing – original draft, Writing – review & editing. NF: Writing – review & editing. NK: Conceptualization, Writing – original draft, Writing – review & editing. PZ: Conceptualization, Writing – original draft, Writing – review & editing. WÅ: Conceptualization, Writing – review & editing. SG: Writing – review & editing. ZW: Conceptualization, Writing – review & editing. NB: Funding acquisition, Writing – review & editing.

## Footnotes

^1^Website. https://www.covid19dataportal.it/.
